# Implications of long-term low-fidelity *in situ* simulation in acute care and association with a reduction in unexpected cardiac arrests: A retrospective research study

**DOI:** 10.1371/journal.pone.0213789

**Published:** 2019-03-12

**Authors:** Chih Jung Wang, Su Yueh Lin, Sheng Han Tsai, Yan Shen Shan

**Affiliations:** 1 Division of Trauma, Department of Surgery, National Cheng Kung University Hospital, Tainan, Taiwan; 2 Department of Nursing, National Cheng Kung University Hospital, Tainan, Taiwan; 3 Department of Internal Medicine, National Cheng Kung University Hospital, Tainan, Taiwan; 4 Division of General Surgery, Department of Surgery, National Cheng Kung University Hospital, Tainan, Taiwan; 5 Institute of Clinical Medicine, College of Medicine, National Cheng Kung University, Tainan, Taiwan; Nord University, NORWAY

## Abstract

In situ simulation is a new tool for building teamwork during crisis. However, only a few studies have discussed the long-term effects of regular in situ simulations. To better understand these effects, this study retrospectively analyzed the effect of regular (twice a month over a four-year period) in situ simulations in the National Cheng Kung University Hospital acute care ward, which provides care for patients with acute illnesses and requires admission during an emergency room visit. The simulations were held in a real clinical environment using a low-fidelity mannequin and the trainees involved in the simulations were the medical staff of the acute care ward. In this study, we review the effects of such long-term simulations with respect to team performance based on the Ottawa global rating scale (GRS) and incidences of urgent intubation and unexpected cardiac arrest. Our results revealed that among the 84 simulations that were conducted during the study period, 42 could be categorized as “high performance” and the remaining 42 as “low performance” based on the team’s Ottawa GRS. Further, the seniority of nurse leaders and exposure of nurses to repeated simulations did not have any effect on performance. However, although regular simulations did not have any effect on the number of urgent intubations, they caused a marked decrease in the number of unexpected cardiac arrests. The current study did not show that repeated, low-fidelity, regular in situ simulations improve team performance in simulations based on Ottawa GRS, but it was associated with a reduction in the unexpected cardiac arrest rate in the acute care ward. Our results support the use of in situ simulations in acute care wards as an educational tool for first-line caregivers.

## Introduction

The treatment of patients suffering from unexpected clinical deterioration in hospitals is an important healthcare issue and positive patient outcomes require effective resuscitation in the early stages of deterioration [1–4]. Studies have found that a reduction in the rate of occurrence of cardiopulmonary arrests outside of intensive care units depends on the ability of first-line caregivers to know when to call for the help of the rapid response team [1, 5, 6]; however, not all staff can accurately identify the clinical cues in critical situations that enable them to determine the necessity calling for help [7].

In such situations, studies have found simulations to be effective tools in teaching principles of crisis resource management (CRM), including communication, decision-making, situation awareness, leadership, and teamwork [3, 8–11]. Simulated crises allow trainees to practice CRM skills in a safe environment. One specific type of simulation that is commonly used in medical institutions is the *in situ simulations* (ISS), in which scenarios are conducted in an actual clinical environment with actual clinical team members. Such simulations can promote both team and unit level learning[11] and allow trainees to evaluate and improve their teamwork and clinical practices through debriefing [3, 12, 13]. Despite the proven benefits of ISS, only a few studies discuss the long-term effects of regularly using ISS within one unit and the impact of simulation-based CRM training on clinical outcomes, because ISS is a relatively new approach [3, 14, 15]. Most studies focus on the performance of simulations [11, 14]. Although one study discussed the clinical effects of regular ISS, it focused on the medical emergency team rather than on the staff in wards [3]. To date, no study has discussed the effectiveness of using regular ISS in acute care wards. To address some of the gaps in understanding, we studied the effects of regular low-fidelity ISS(a full-body mannequin for cardiopulmonary resuscitation, not a high fidelity simulator) in a local acute care ward since 2012. In this study, we hypothesized that regular ISS would improve team performance. Further, we clarified the impact of using ISS in acute care wards on the incidence of urgent intubation and unexpected cardiac arrest.

## Material and methods

This retrospective study evaluated the effectiveness of using long-term, regular ISS in an acute care ward. Our institutional review board approved this study (A-ER-105-323). The study was conducted at National Cheng Kung University Hospital, a 1200-bed tertiary medical center in South Taiwan that has approximately 84,000 emergency room (ER) visits annually. In 2011, the hospital opened a new 44-bed acute care ward to provide better care for ER patients requiring admission: all admitted patients are acutely ill, and some of the cases are complicated. Despite implementing various efforts to ensure better care for critical patients in this ward, the hospital does not have a rapid response team, which implies that nursing staff must help resuscitate patients in critical events. Patients are primarily cared for by young residents and interns, who often lack experience in managing critical events and organizing a medical team. Therefore, to enhance their communication and teamwork skills and help medical teams implement these skills in their routine work, we started an ISS program in the acute care ward in July 2012 [16].

### Low-fidelity in situ simulations

**Fig 1:**
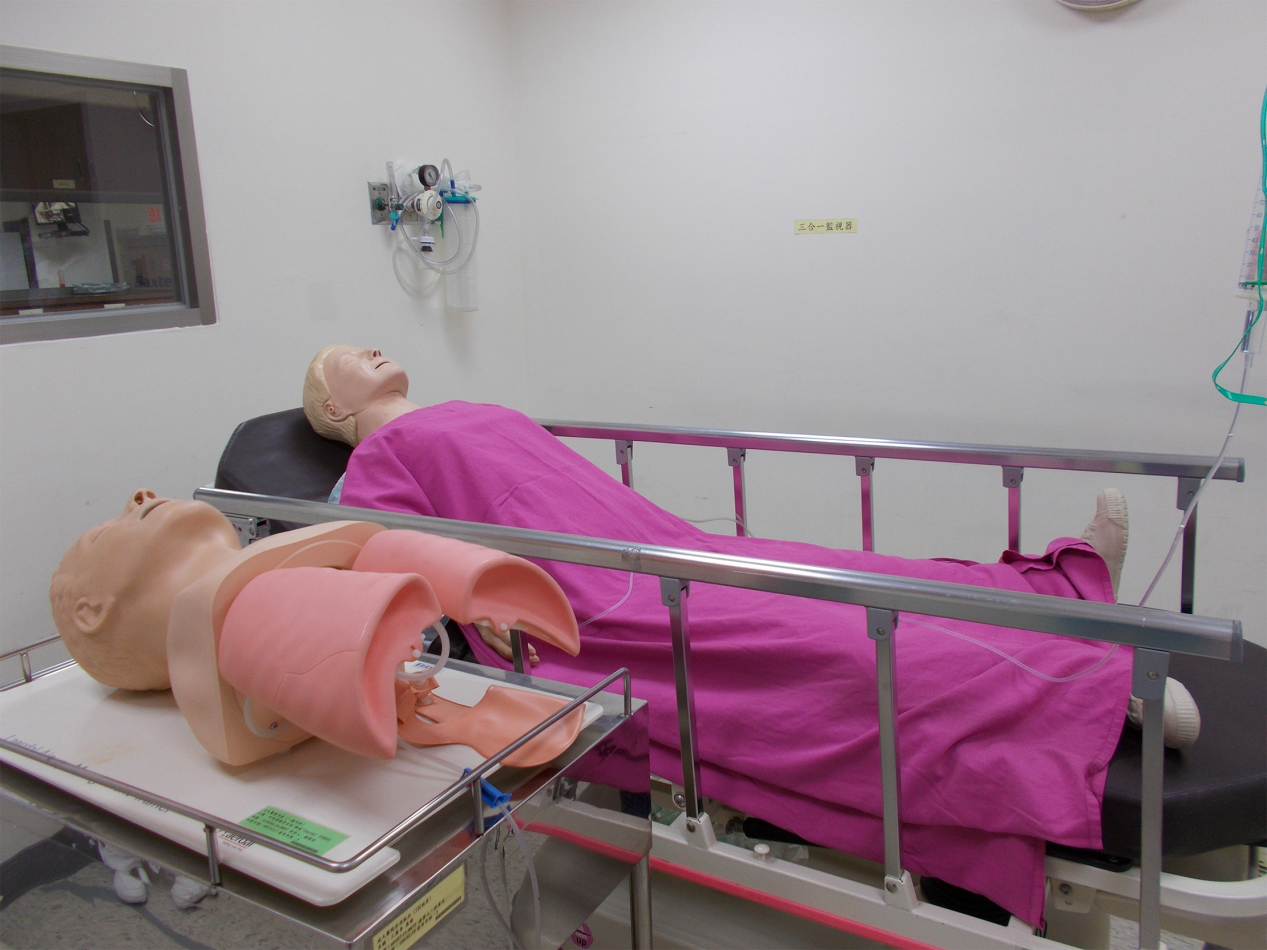
Use of a low-fidelity mannequin used in in situ simulations.

Twice a month, ISS sessions were conducted during the morning shift change. The participating staff members were alerted one day before each simulation. Instead of using a high-fidelity mannequin, we used low-fidelity equipment, including one full body mannequin (i.e., Anne), one airway management trainer, and a DART ECG (electrocardiogram) simulator, to conduct the scenario. Despite the low-fidelity equipment, we established high psychological fidelity through induction of a multi-task scenario. A rubber tube embedded in the mannequin’s arm served as a vein for the fluid and drug administration. The participating nurse had to draw and push medication through this “vein.” Depending on the scenario, trainees were required to complete tasks that included patient evaluation, intubation, chest compression, defibrillation, resuscitation, and drug administration. As part of the simulation, they had to survey the patient, interpret vital signs and ECG rhythm on the ECG simulator, prepare equipment and medication for intubation, set intra-venous lines for fluid administration, and administer medication (Fig 1).

The image depicts both a full-body mannequin used for cardiopulmonary resuscitation and an airway management trainer that simulates the sequence of oral intubation.

Each simulation included an intern, two junior residents, and four nurses; other members of the medical team were not involved in the simulation and acted as observers. One of the physicians was selected as the leader and was required to make all relevant critical decisions. Since young physicians were often unfamiliar with the nurses working in the acute care ward, which made it difficult for them to organize and lead the team, we designated one nurse, typically the most experienced one, to help the physician assign tasks to other nurses, communicate with physicians to confirm the priority of tasks, and monitor the situation. The nurse leader who facilitated nurse-physician cooperation during the simulation, was a key member of the team.

Since residents and interns are moved to different wards every month, only nurses could benefit from repeated ISS training; therefore, our study focused mainly on the nursing staff. Our primary goal was to teach them teamwork by conducting various on-the-job situations, including early call for help, iSBAR (identification, situation, background, assessment, recommendation), direct communication, closed loop communication, cross monitoring, situation awareness, and resource management [17].

The ISS program focused on the development of CRM skills. We evaluated team performance using the Ottawa Crisis Resource Management Global Rating Scale (Ottawa GRS)[18], which is a well-known tool for evaluating CRM skills. The Ottawa GRS uses a 7-point Likert scale to assess performance in five categories (leadership skills, problem solving skills, situational awareness skills, resource utilizations skills, and communication skills) and an overall performance score [19, 20]. Therefore, the highest score was 42 and lowest was seven. The Ottawa GRS can be used to evaluate both individual and team performance [18, 19]. Two fixed trainers, a head nurse and an attending physician, helped instruct each simulation, conduct the scenario, debrief the participants, and rate each performance. We focused on overall team performance rather than on individual performance when scoring with Ottawa GRS. Each ISS was completed in 30 minutes, and comprised of a briefing (5 minutes), the simulation (10 minutes), and a debriefing (15 minutes). During the debriefing, we mainly focused on CRM skills such as communication and teamwork[21]. The debriefing started with the trainee providing feedback on either overall team performance or the performance of a team member, and the trainer guided the discussion to keep the team focused on CRM skills and helped them think about using CRM skills to improve team or personal performance.

### Outcomes

As described earlier, the Ottawa GRS scores were the primary outcome[18]. In addition, we also reviewed incidences of urgent intubation and unexpected cardiac arrest requiring CPR in the acute care ward between 2011 and 2015.

### Statistical analysis

We analyzed continuous data using the Student t-test or the Kruskal-Wallis Test as appropriate, and categorized data using the chi-square test. We suspected that implementing ISS in acute care wards would not immediately affect the clinical outcomes; therefore we defined the period from 2011 to 2012,which constituted the first half year after ISS implementation as the early study (pre-intervention) period and the period from 2013 to 2015 as the late study (post intervention) period, and examination as the secondary aim.

## Results

We conducted 84 ISS sessions during the study period. The participating staff members included 53 nurses, of whom 16 served as simulation leaders at least once. We divided the ISS sessions into the following two groups based on the Ottawa GRS score: high-performance sessions, which scored more than 50%, and low-performance sessions, which scored less than 50%.

We determined that there were no significant differences between the two groups in terms of leaders’ seniority, members’ seniority, leaders’ simulation experience, and members’ simulation experience (Table 1). Further, we did not observe any difference in the number of incidences of urgent intubation. However, we found that the incidence of unexpected cardiac arrest decreased from 0.04% to 0.02% during the later period (p = 0.09) (Table 2).

**Table 1:** Characteristics of high performance and low performance groups.

	High Performance N = 42		Low Performance N = 42		
	Mean	SD	Mean	SD	p-Value
Ottawa GRS					
Overall	5.85	0.7	4.65	0.72	<0.0001
Leadership	6.02	0.7	4.68	0.58	<0.0001
Problem solving	6.06	0.6	4.76	0.73	<0.0001
Situation awareness	5.76	0.7	4.75	0.59	<0.0001
Resource utilization	6.24	0.6	4.68	0.63	<0.0001
Communication	6.24	0.6	4.75	0.91	<0.0001
Simulation leader experience of lead nurse	3.83	2.8	3.29	2.73	0.3666
Simulation experience of team members	4.41	2.1	4.51	1.95	0.8314
Leader’s seniority (year)	10.78	7.8	9.04	7.21	0.2921
Member’s seniority (year)	3.74	2.3	3.41	2.61	0.5426

Ottawa GRS, Ottawa Global Rating Scale; SD, standard deviation.

**Table 2:** Comparison of emergent intubations and CPR between different periods.

	Periods		
	Period 1 (2011–2012)	Period 2 (2013–2015)	p-Value ^a^
Intubation N (%)	44 (0.15)	56 (0.12)	0.3
CPR N (%)	12 (0.04)	9 (0.02)	0.09

CPR, cardiopulmonary resuscitation.

^a^ Chi-Square Test.

A comparison of periods 1 and 2 showed that there was no difference in the incidences of urgent intubation. The incidences of unexpected cardiac arrest decreased from 0.04% to 0.02% (p = 0.09) (Table 2).

## Discussion

This study evaluated the effects of regular ISS in an acute care ward over a period of four years. Our findings reveal that nurses’ seniority, and experience with ISS had no effect on Ottawa GRS score. This result differs from that of previous studies, according to which repeated exposure to simulations improved performance. [13, 19, 22] There are several reasons for the differences in the findings of our study from those of earlier studies. First, this was not a short-duration education course in which the trainee received training and evaluation in one day [12, 19]. In this study, ISS was a continued education tool in the acute care ward; we held the simulation twice a month. This may attenuate the effect of each simulation. Some previous studies found that repeated exposure to a simulation more than two times did not guarantee continued improvement [19, 20, 23]. They hypothesized that most observed non-technique improvement occurred in the early phase of simulation training. Second, we did not evaluate the effect of observation in this study. One of the advantages of ISS is that medical staff members in the unit who are not participating in the simulation can act as observers. They have the opportunity to learn critical resuscitation procedures while increasing their teamwork skills by observing the simulation and being involving in debriefing, even when they are not immediately involved in the simulation. The observers can learn CRM skills along with the active participants in the simulation [24, 25]. This study may have not actually evaluated the effect of ISS by not evaluating the effect of observation. In addition, unlike in earlier studies, physicians did not receive ongoing training in ISS and nurses participated in more than one ISS session in our study. Since residents and interns switch wards every month, we could not provide them with ongoing simulation training. This may have negatively affected team performance.

Our results reveal that simulations do not have to be high fidelity to impact clinical outcomes. High-fidelity simulations can be expensive, and the effective use of low-fidelity equipment makes the implementation of ISS training programs easy [26]. However, by setting high environmental and psychological fidelity, in the situation of multi-tasking, the tension of the simulation was just like a real event. Thus, low-fidelity equipment simulations can still provide vital skill building opportunities to trainees [26–29]. For institutions with limited financial resources, low-fidelity ISS is an effective tool to start ISS training [22, 26].

ISS also has several disadvantages [30, 31], the most important of which is asking medical staff to step away from their daily work, particularly within the clinical environment. To minimize this inconvenience, we condensed each simulation to 30 minutes twice a month. During the 15 minutes of debriefing, we focused our discussion on one or two soft skills, including teamwork. Keeping the debriefing short not only saved time but also helped trainees to focus on one skill at a time; through repeated training, they can accumulate experience in many skills. Further, we rarely had to postpone the sessions because of clinical emergencies. In our experience, long-term, short-duration simulations are feasible and sustainable.

Our results showed regular ISS in an acute care ward to have a significant impact on the rate of unexpected cardiac arrest, which is in line with the findings of previous studies on patient outcomes and a few studies that revealed how simulation-based CRM training and caregiver education can improve patient outcomes [1, 3, 5]. Identifying acute physiological deteriorations in acutely ill patients and calling for help can reduce the cardiac arrest rate [32]. Our scenarios helped teach nurses to recognize the signs of deterioration as well as the importance of early reporting and communicating with physicians [3], and ISS instilled a culture of teamwork and communication [33]. Regular ISS is an effective educational tool for first-line caregivers in the acute care ward.

This study has some limitations. First, this was a retrospective study; therefore, we collected Ottawa GRS scores during the study period and data on patient outcomes later. Because of this schedule, some essential data could have been missed. In addition, the study period was four years; over such a long period, the rater criteria for team performance may undergo change. Further, we do not have video recordings of all the sessions, so we cannot reevaluate performance based on common criteria. The decrease in incidences of cardiac arrest may have been the result of improved communication between nurses and physicians and the increase in the use of palliative care. While we lacked any palliative care data, the incidences of intubation remained the same during the study period, which suggests that palliative care did not increase during the period. Finally, the personal experiences of the nurse leader (i.e., in CPR and/or critical care), may affect team performance, however we did not collect any data to determine this effect.

## Conclusion

To the best of our knowledge, this is the first study to analyze the impact of long-term regular ISS in an acute care ward. We determined the effects of regular ISS on team performance and revealed that ISS reduced the incidence of emergent intubation and unexpected cardiac arrest. Based on our experience, low-fidelity ISS is a feasible tool for continuous education to build teamwork and teach clinical staff crisis resource management skills. Since the current study is a retrospective one, we did not address the issues like critical patient care quality and comfort care. Further prospective research is necessary to determine the effect of long-term ISS in the health care unit.

## Supporting information

S1 File Open access anonymized data for current study. (XLSX)
